# Comparative clinico-haematological analysis in young Zebu cattle experimentally infected with *Trypanosoma vivax* isolates from tsetse infested and non-tsetse infested areas of Northwest Ethiopia

**DOI:** 10.1186/s13028-015-0114-2

**Published:** 2015-05-19

**Authors:** Shimelis Dagnachew, Melkamu Bezie, Getachew Terefe, Getachew Abebe, J. David Barry, Bruno M. Goddeeris

**Affiliations:** Department of Pathology and Parasitology, College of Veterinary Medicine and Agriculture of Addis Ababa University, P.O. Box: 34, Debre Zeit, Ethiopia; Food and Agriculture Organization of the United Nations, Addis Ababa, Ethiopia; Wellcome Trust Centre for Molecular Parasitology, College of Medicine, Veterinary and Life Sciences, University of Glasgow, 120 University Place, G12 8TA Glasgow, UK; Department of Biosystems, Faculty of Bioscience Engineering, Catholic University of Leuven, 30 bus 2456, B-3001 Heverlee, Belgium; Faculty of Veterinary Medicine, University of Gondar, P.O. Box: 196, Gondar, Ethiopia

**Keywords:** Clinical findings, Haematological analysis, Northwest Ethiopia, *Trypanosoma vivax*, Zebu cattle

## Abstract

**Background:**

Ethiopia, particularly in the Northwest region, is affected by both tsetse and non-tsetse fly transmitted trypanosomosis, with significant impact on livestock productivity. The aim of this study was to determine and compare clinical findings and haematological values between experimental infections induced by *Trypanosoma vivax* isolates from areas of either transmission mode. Sixteen young (aged between 6 and 12 months) Zebu cattle (*Bos indicus*), purchased from a trypanosome-free area and confirmed to be trypanosome-negative, were randomly assigned into four groups of four animals. Groups 1, 2 and 3 were infected with an isolate from a tsetse infested or one of two isolates from a non-tsetse infested area, and group 4 was a non-infected control. All animals in the infected groups were inoculated intravenously with 2 × 10^6^ trypanosomes from donor animals. The experimental animals were monitored for eight consecutive weeks post infection for clinical signs, parasitaemia and haematological changes in packed cell volume (PCV), haemoglobin concentration (Hgb), total red blood cell (RBC) and white blood cell (WBC) counts, differential WBC count and blood indices (mean corpuscular volume [MCV], mean corpuscular haemoglobin and mean corpuscular haemoglobin concentration).

**Results:**

Infection was characterized by reduced feed intake, weakness, pyrexia, parasitaemia, rough hair coat, enlarged prescapular lymph nodes, lacrimation, weight loss, pallor mucus membrane and dehydration. Body weight loss in all infected groups was significantly higher than in the non-infected control. Similarly, body weight loss was higher (*P* < 0.001) in animals infected with the tsetse infested isolate than with the non-tsetse infested isolates. The mean PCV, Hgb, total RBC and WBC counts were lower (*P* < 0.001), and mean MCV was higher (*P* = 0.01) in all infected groups than in non-infected control animals at different time points during the study period. Except for minor variations in haematological values, the overall changes were similar in all infected groups.

**Conclusion:**

Clinical signs and significant reduction in haematological values in the infected groups indicated the pathogenicity of the *T. vivax* parasites. Pathogenicity of *T. vivax* from the non-tsetse infested area can be considered as nearly as important as that of its counterpart derived from the tsetse infested area.

## Introduction

Trypanosomosis is a parasitic disease caused by different species of flagellated protozoa belonging to the genus *Trypanosoma* which inhabit the blood, various body tissues and fluids of vertebrate hosts. However, the extent of tissue invasion varies among the different species of the parasite [1, 2]. The disease is frequently fatal and is a serious constraint to agricultural production in large parts of sub-Saharan Africa, exhibiting direct impacts on livestock productivity, livestock management and human settlement, and indirect impacts on crop agriculture and human welfare [3]. Trypanosomosis is transmitted cyclically by tsetse flies; however, some species of trypanosome such as *T. vivax* are also transmitted mechanically, by other biting flies, such as several tabanids [4]. *T. vivax* presents a short and simple life cycle in contrast with *T. brucei* and, to a lesser extent, with *T. congolense* [5]. This simpler life cycle is thought to have enabled *T. vivax* to adapt to different vectors and hosts, possibly explaining why it has emerged rapidly in South America and non-tsetse infested regions of Africa [6, 7]. In Ethiopia, trypanosomosis is mainly prevalent in the northwest and southwest areas of the country [8–11], where *T. vivax* and *T. congolense* have been identified as the prevalent species. *T. vivax* is distributed in both tsetse infested and non-tsetse infested areas, while *T. congolense* is restricted to tsetse infested areas. Both species of trypanosome affect a large number of domestic animals, and mainly cattle, which are integral to crop production in Ethiopia. As opposed to prevalence, the comparative pathological impact of *T. vivax* parasites which is transmitted cyclically by tsetse fly and mechanically by other biting flies is not well known.

The pre-patent period of bovine trypanosomosis is usually 1 to 3 weeks, depending on the virulence of the infecting trypanosome, the infective dose and the immune status of the host. Symptoms associated with trypanosomosis include pallor of the mucous membranes, enlargement of lymph nodes and spleen, weakness, loss of condition, abortion and reduced milk production [12]. The evaluation of haematological values is of paramount importance in determining the health status of animals [13]. The main haematological changes observed in natural cases of bovine trypanosomosis are anaemia associated with decrease in packed cell volume (PCV), haemoglobin (Hgb) and total red blood cell (RBC) counts, and severe leukopenia [14, 15]. The severity of anaemia which occurs in infection can be related to differences in virulence among trypanosome strains and species, and factors associated with the host like age, breed, and nutritional status of infected animals [14].

Isolates of *T. vivax* from different parts of the world have been shown to differ in pathogenicity and infectivity for laboratory rodents and in requirements for cultivation *in vitro* [7]. However, despite the severe disease it causes, *T. vivax* remains largely unstudied in both tsetse and non-tsetse infested areas. Moreover, less is known about the biology of *T. vivax* than about that of *T. brucei* and *T. congolense* in East Africa. It is generally believed that East African strains of *T. vivax* are less pathogenic and cause a chronic form of infection; however, occasional outbreaks of *T. vivax* acute infection have been reported in Kenya and Uganda [16]. For *T. vivax*, neither the possible occurrence of such an acute disease form, nor pathogenic impact at large, has been investigated in Ethiopia. Furthermore, information on the impact of *T. vivax* infections on haematological values is scarce. To attain better understanding of the pathogenicity of *T. vivax* in northwest Ethiopia, the present study determined and compared clinical findings and haematological values induced by three *T. vivax* isolates, one from tsetse infested area and two from non-tsetse infested area in experimentally infected young Zebu cattle.

## Materials and methods

### Experimental study site

The study was conducted, from March to May 2014, in a fly proof animal facility recently established by GALVmed at the premises of the College of Veterinary Medicine and Agriculture of Addis Ababa University. The area is located about 45 km Southeast of Addis Ababa at 9°N latitude and 40°E longitude and altitude 1850 m above sea level.

### Experimental animals

Sixteen young Zebu (*Bos indicus*) cattle (six male, ten female) aged between 9 and 12 months were purchased from Debre Berehan, which is located in a trypanosome free area of northcentral highland Ethiopia. Before transportation, animals were treated with long acting oxytetracycline (Alamycin LA, Norbrook, Ireland). On arrival at the experimental study site, the animals were ear-tagged and screened for haemoparasites and other internal and external parasites. Animals were dewormed with albendazole (Albenda-QK, Chengdu Qiankun, China) and ivermectin (Ivermectin 1 %, Chengdu Qiankun, China), to control internal and external parasites. Prior to the experiment, the animals were acclimatized for one month to the new environment, handling and feeding conditions.

### Feeding and animal management

Throughout the experimental period, animals were fed *ad libitum* with grass hay and water, with supplements of concentrates and mineral licks. All protocols and procedures of the handling of animals were according to the international guiding principles for biomedical research proposed by the Council for International Organizations of Medical Sciences [17] and ARRIVE guidelines [18]. The research was authorized by the Animal Research and Ethics Review Committee of the College of Veterinary Medicine and Agriculture of Addis Ababa University (Permit No: VM/ERC/003/04/013). At the end of the experiment, all of the infected animals were euthanized by jugular vein injection of an overdose of sodium phenobarbitol. In addition, any animals with the severe clinical manifestations of recumbence and PCV below 15 % were euthanized using the above procedure.

### Experimental groups

The experimental animals were divided randomly into four equal groups of four animals per group. Group 1 (TT) were infected with *T. vivax* isolated from a tsetse infested area; Group 2 (NT1) with *T. vivax* isolated from a non-tsetse infested area; Group 3 (NT2) with another strain of *T. vivax* isolated from a non-tsetse infested area; and Group 4 (NIC) was the non-infected control. Each group was kept in a separate pen. Attempts to induce infections with a second strain isolated from a tsetse infested area were unsuccessful, restricting the experiment to only one strain of this type.

### Trypanosome challenge

The *T. vivax* parasites were originally isolated from naturally infected cattle from the Jabitehenan district of Birsheleko area (ETBS2-Ethiopia Birsheleko isolate 2-tsetse infested area) and Bahir Dar Zuria district (ETBD2 and ETBD3-Ethiopia Bahir Dar isolate 2 and 3-non-tsetse infested area). From these animals, stabilates were prepared and cryopreserved in liquid nitrogen (−196 °C). The isolates were confirmed as pure *T. vivax* by polymerase chain reaction [19] and microsatellite analysis [20]. The cryopreserved stabilates were propagated in three healthy donor calves. The donor animals were examined daily for parasitaemia using the rapid “matching” method [21]. All experimental animals except the non-infected controls received intravenously 2 mL of blood taken from donor animals at a parasitaemia level of 10^6^ trypanosomes/mL.

### Clinical and parasitological examinations

Animals were examined daily for clinical parameters at their pen throughout the study period. Rectal temperature was taken twice weekly, in the morning, using a digital thermometer. Body weight of each animal was measured weekly on an electronic cattle weighing scale (TAL-TEC®, South Africa). Body weight gain was calculated weekly, relative to the day 0 body weight. Blood samples were examined for the presence of trypanosomes daily until the detection of parasites, then weekly, using the standard parasitological methods of wet blood smear microscopy and the buffy coat technique [22]. All the sampling protocols was designed according to ARRIVE guidelines that minimize influence on the results of the research.

### Haematological analysis

About 5 mL of blood was collected weekly during the study period from the jugular vein of all experimental animals using ethylene diamine tetra acetic acid (EDTA) coated vacutainer tubes. Haematological parameters measured included: PCV, Hgb concentration, RBC count, erythrocyte indices (mean corpuscular volume (MCV), mean corpuscular haemoglobin (MCH) and mean corpuscular haemoglobin concentration (MCHC)), and total and differential white blood cell (WBC) count. PCV was measured by the haematocrit centrifugation technique using a Hawksley microhaematocrit reader. Total RBC and WBC counts were carried out manually using the improved Haemocytometer. Hgb concentration was measured by the Sahili’s Acid-Haematin method. Erythrocyte indices were also calculated from the above haematological values using the following formulas.

$$ \mathrm{M}\mathrm{C}\mathrm{V}=\mathrm{P}\mathrm{C}\mathrm{V}\left(\%\right) \times 10/\mathrm{R}\mathrm{B}\mathrm{C}\ \mathrm{count} $$

$$ \mathrm{MCHC}=\mathrm{H}\mathrm{g}\mathrm{b}\ \mathrm{con}. \times 100/\mathrm{P}\mathrm{C}\mathrm{V} $$

$$ \mathrm{M}\mathrm{C}\mathrm{H}=\mathrm{H}\mathrm{g}\mathrm{b}\ \mathrm{con}. \times 10/\mathrm{R}\mathrm{B}\mathrm{C}\ \mathrm{count} $$

Thin blood smears were stained with Giemsa’s for differential WBC counts, which were based on 100 cells per slide according to their staining reactions; shape of the nucleus, and presence or absence of granules in their cytoplasm [13]. The absolute numbers of leukocytes, eosinophils, lymphocytes, neutrophils, basophils and monocytes per milliliter of blood were obtained by using the differential white cell count percentages and the total leukocytes count.

### Data analysis

All data were entered into an Excel spread sheet and imported into SPSS version 20 statistical software. Descriptive statistics were used to describe the data. Differences in haematological variables, rectal body temperature and body weight loss measured between groups were assessed by a one-way ANOVA. The significant level was set at (*P* < 0.05).

## Results

### Clinical findings and appearance of parasitaemia

**Fig. 1:**
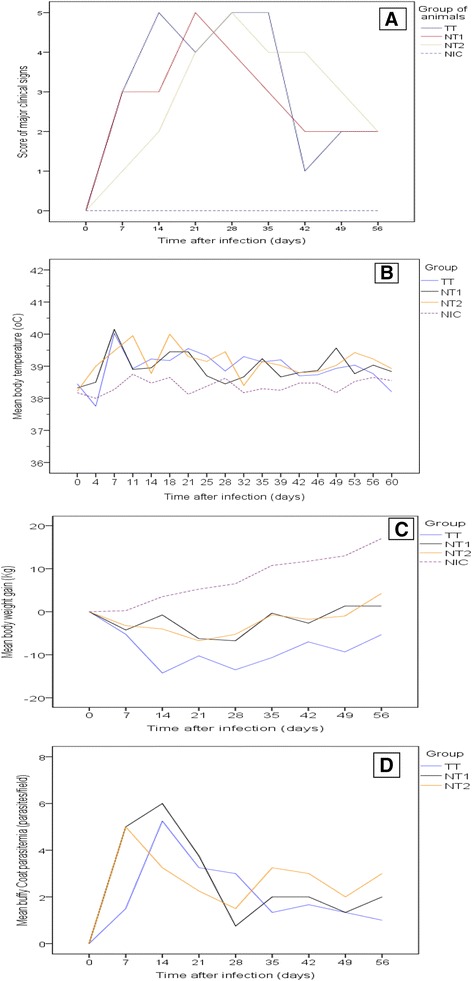
Clinical findings and appearance of parasitaemia in young zebu cattle experimentally infected with *T. vivax* isolates from tsetse-infested (TT) and non-tsetse infested (NT1 and NT2) areas and non-infected control (NIC) groups during the study period. **a** Score of major clinical signs (reduction in feed intake, enlarged lymph nodes, rough hair coat and pallor of mucus membranes), (**b**) Mean rectal temperature in °C, (**c**) Mean body weight gain in kg, (**d**) Mean waves of parasitaemia (score per microscopic field)

All the infected cattle developed clinical trypanosomosis, which was characterized by a variety of clinical signs. Reduced feed intake, weakness, fever, rough hair coat, enlarged superficial lymph nodes, congested mucus membranes, lacrimation and weight loss were early clinical findings, whereas pallor of mucus membranes, dehydration, and emaciation were predominant later in infection. The major clinical signs (reduced feed intake, enlarged lymph nodes, rough hair coat and pallor of mucus membranes) observed at each time point were added together and scored accordingly to the experimental groups, as indicated in Fig. 1a. Signs observed less frequently included dullness, diarrhoea, corneal opacity and recumbence. Two animals (one from the TT and the other from the NT1 group) showed severe clinical signs of recumbence and PCV below 15 % and hence were euthanized on post infection day (PID) 30. The mean rectal temperatures of infected groups (39.1 °C ± 0.64, 39.2 °C ± 0.70, 39.2 °C ± 0.70 for TT, NT1, NT2, respectively) were significantly higher (*P* < 0.001) than for the control group (38.39 °C ± 0.30). However, no significant difference was found among infected groups. The temperature of infected animals started rising from the PID 4, coincident with the appearance of parasitaemia, and then fluctuated throughout the study period (Fig. 1b). The highest mean temperature recorded was 40.15 °C, from the NT1 group on PID 7. Infected groups significantly lost body weight, while the control group steadily gained weight (*P* < 0.001). Body weight loss was significantly higher (*P* < 0.001) in group TT than in groups NT1 and NT2. This decrease in body weight of infected animals was marked and progressive between PIDs 0 and 28. Thereafter, weight tended to stabilize, but remained significantly (*P* < 0.001) below that of control animals until the termination of the experiment (Fig. 1c).

Parasitaemia was detected by PID 4 in animals infected with *T. vivax* isolates from the non-tsetse infested area and by PID 7 in animals infected with the tsetse infested isolate. Animals in group NT2 showed early peak parasitaemia on PID 7, as opposed to the TT and NT1 groups, which showed peak parasitaemia on PID 14. Animals remained trypanosome positive with fluctuating parasitaemia throughout the experimental period. High fluctuations were seen in animals infected with non-tsetse isolates, while parasitaemia dropped uniformly after its initial peak for the tsetse infested isolate infected group (Fig. 1d). In group NT2, despite sharp decline after the initial peak on PID 7, the parasitaemia persisted, reaching its highest level from PID 35 to 56.

### Haematological findings

**Fig. 2:**
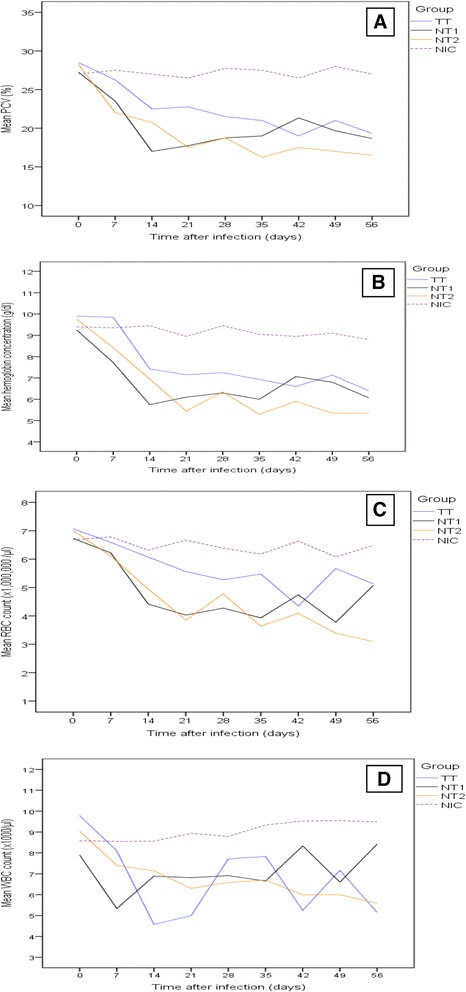
Mean haematological values in young Zebu cattle experimentally infected with *T. vivax* isolates from tsetse infested (TT) and non-tsetse infested (NT1 and NT2) areas and non-infected control (NIC) groups during the study period. **a** Mean PCV values in percent, (**b**) Mean haemoglobin concentration in g/dl, (**c**) Mean total RBC count in × 10^6^ cells/μl, (**d**) Mean total WBC count in × 10^3^ cells/μl

The overall mean haematological changes of all experimental animals during the study period are summarized in Tables 1 and 2. Likewise, the haematological changes of all experimental animals at different time points of the study period are indicated in Fig. 2a–d. Significant decrease (*P* < 0.05) of mean PCV, Hgb concentration, total RBC and WBC counts was detected in all infected groups compared with the non-infected control group. Mean PCV values of the infected groups decreased gradually, attaining significant reduction by PID 14 (Fig. 2a). All infected groups showed significant decrease (*P* < 0.001) in mean PCV as compared with the non-infected control group. In addition, PCV values of group NT2 were significantly lower (*P* = 0.001) than those of group TT. Infected groups had significant reduction (*P* < 0.001) in mean Hgb concentration as compared with the non-infected control group (Fig. 2b). Moreover, the mean Hgb concentration value of group NT2 was significantly lower (*P* = 0.030) than that of group TT. Reductions in Hgb concentration for groups TT and NT1 started to reach significance at PID 14, but did so in group NT2 only by PID 21 and persisted until the end of the study. For group NT1, Hgb concentration was not significantly reduced (*P* = 0.062) on PID 42.

**Table 1:** Mean haematological values during the study period (mean of recordings of 8 consecutive weeks) in young Zebu cattle experimentally infected with *T. vivax* isolates from tsetse infested (TT) and non-tsetse infested (NT1 and NT2) areas, and non-infected control (NIC)

Hematological values	Group	Mean ± SD	95 % CI for mean
PCV values (%)	TT	22.72 ± 3.55^a^	21.44-24.0
	NT1	20.41 ± 3.67^ab^	19.08-21.73
	NT2	19.39 ± 4.59 ^b^	17.84-20.94
	NIC	27.19 ± 2.05^c^	26.5-27.89
Hgb concentration (g/dl)	TT	7.73 ± 1.13^a^	7.22-8.25
	NT1	6.82 ± 1.34^ab^	6.34-7.30
	NT2	6.53 ± 1.86a^b^	5.90-7.17
	NIC	9.17 ± 0.68^c^	8.94-9.40
RBC count (×10^6^/μl)	TT	5.75 ± 0.91^a^	5.42-6.08
	NT1	4.85 ± 1.20^ab^	4.41-5.28
	NT2	4.54 ± 1.46^b^	4.05-5.04
	NIC	6.47 ± 0.64^c^	6.25-6.68
MCV (fl)	TT	42.29 ± 3.77^a^	41.02-43.57
	NT1	43.25 ± 7.08^a^	40.70-45.80
	NT2	44.34 ± 7.83^a^	41.69-47.00
	NIC	39.64 ± 2.98^b^	38.57-40.72
MCH (pg)	TT	13.46 ± 1.34^a^	12.98-13.95
	NT1	14.40 ± 2.23^a^	13.60-15.21
	NT2	14.72 ± 2.36^a^	13.92-15.52
	NIC	14.25 ± 1.24^a^	13.83-14.68
MCHC (g/dl)	TT	33.97 ± 2.36^a^	33.12-34.82
	NT1	33.38 ± 1.61^a^	32.80-33.96
	NT2	33.39 ± 2.56^a^	32.52-34.26
	NIC	33.74 ± 1.34^a^	33.28-34.19
WBC count (×10^3^/μl)	TT	6.78 ± 2.51^a^	5.87-7.68
	NT1	7.04 ± 2.03^a^	6.31-7.77
	NT2	6.75 ± 1.57^a^	6.21-7.28
	NIC	9.03 ± 1.17^b^	8.64-9.43

In each parameter of variables, superscripts with different letters indicate significant difference between values at *P* < 0.01

**Table 2:** Mean differential WBC counts during the study period (mean of recordings of 8 consecutive weeks) in young Zebu cattle experimentally infected with *T. vivax* isolates from tsetse infested (TT) and non-tsetse infested (NT1 and NT2) areas, and non-infected control (NIC)

Haematological values	Group	Mean ± SD	95 % CI for mean
Lymphocyte count (×10^3^/μl)	TT	4.11 ± 1.82^a^	3.46-4.77
	NT1	4.11 ± 1.07^a^	3.72-4.50
	NT2	4.68 ± 1.44^a^	4.19-5.16
	NIC	5.54 ± 0.79^b^	5.28-5.81
Monocyte count (×10^3^/μl)	TT	0.59 ± 0.35^a^	0.46-0.71
	NT1	0.57 ± 0.28^a^	0.47-0.68
	NT2	0.42 ± 0.20^a^	0.35-0.49
	NIC	0.49 ± 0.20^a^	0.42-0.56
Neutrophil count (×10^3^/μl)	TT	1.84 ± 1.00^a^	1.48-2.20
	NT1	2.14 ± 1.44^a^	1.62-2.67
	NT2	1.50 ± 0.75^a^	1.24-1.75
	NIC	2.60 ± 0.77^b^	2.34-2.86
Eosinophil count (× 10^3^/μl)	TT	0.20 ± 0.22^a^	0.11-0.28
	NT1	0.18 ± 0.11^a^	0.15-0.23
	NT2	0.13 ± 0.14^a^	0.08-0.18
	NIC	0.40 ± 0.23^b^	0.32-0.48
Basophil count (×10^3^/μl)	TT	0.04 ± 0.05^a^	0.02-0.06
	NT1	0.02 ± 0.04^a^	0.00-0.04
	NT2	0.01 ± 0.03^a^	0.00-0.03
	NIC	0.02 ± 0.04^a^	0.01-0.03

In each parameter of variables, superscripts with different letters indicate significant difference between values at *P* < 0.05

All of infected groups had a significantly lower (*P* < 0.001) mean total RBC count as compared with the control group (Fig. 2c). Total RBC counts of groups NT1 and NT2 were also significantly lower (*P* = 0.007, and *P* < 0.001, respectively) than those of group TT. Reductions in mean total RBC count became significant at PID 14 for groups NT1 and NT2, but at PID 21 for group TT, in which the low level persisted until PID 42. However, the reduction was not significant on PID 49 and 56 for groups TT and NT1, respectively. There was a significant increase in mean MCV (*P* = 0.001) in infected groups compared with that of the non-infected group. No significant difference was observed in mean MCH and MCHC values (*P* = 0.056 and 0.581, respectively) between infected and non-infected groups.

The mean total WBC count of infected groups was significantly lower (*P* < 0.001) compared with the non-infected control (Fig. 2d). However, there was no significant difference in mean WBC count between infected groups. The mean differential WBC count (Table 2) indicated that lymphocyte, neutrophil and eosinophil levels were depressed in infected groups (*P* < 0.001). Monocyte count showed insignificant elevation. Mean leukocyte counts in the infected groups began to decline between PID 14 and 21 and remained below the level of the non-infected group until PID 49 and 56 for NT and TT groups, respectively. The basophils counts in all the three infected groups fluctuated within the control levels throughout the experiment.

## Discussion

### Clinical findings and development of parasitaemia

The onset of clinical manifestations coincided with the beginning of parasitaemia and these parameters progressed together. Similar findings were reported by Adeiza *et al*. [23] in Savannah brown goats experimentally infected with *T. brucei* and *T. vivax*. The more pronounced loss in body weight gain of the group infected with *T. vivax* from tsetse area might be due to the more severe dehydration and anorexia manifested. Furthermore, loss of body weight during trypanosome infections in domestic animals has been reported frequently reported by several authors [24, 25], indicating the economic impact of the disease.

The earlier appearance of parasitaemia with the non-tsetse isolates might be associated with the fact that artificial intravenous inoculation is more similar to mechanical transmission of *T. vivax* than to cyclical transmission of tsetse adapted *T. vivax*. Another explanation for the early onset of parasitaemia in the NT infected cattle might be a higher growth rate of the NT parasite. This possibility is supported by the phenomenon in *T. brucei* [26] and *Plasmodium chabaudi* [27] of marked increase in parasitaemia and virulence when these parasites are syringe passaged in rodents. These alterations have been attributed to a lack of a reset mechanism when going through the vector and it seems possible that loss of pleomorphism might apply to mechanically transmitted lines in *T. viax* [28, 29]. Similarly, we have reported previously that non-tsetse transmitted *T. vivax* displayed earlier parasitaemia than did a tsetse transmitted isolate in experimental infection of cattle [30]. In the current work, although parasites appeared in the blood faster in the NT groups, virulence did not seem to be increased compared with the TT group. Similar early onset of parasitaemia was noted in *T. vivax* infected goats by Adeiza *et al*. [23] and Osman *et al*. [31]. The pre-patent period of infection by *T. vivax* is variable, depending on the immune status of the host, virulence of the parasite isolate and the infective dose [12, 7].

### Haematological findings

In the present study, significant decrease in mean PCV, Hgb concentration, total RBC and WBC counts was observed in all infected groups compared with the non-infected group. In agreement with our findings, Maxie *et al*. [32] described pancytopenia, leukopenia, and thrombocytopenia associated with *T. vivax* and *T. congolense* infections of cattle. Similar observations were reported by Osman *et al.* [31] in goats infected with *T. vivax*. The decreases in these parameters indicate a state of anaemia in the infected groups and ascertained the presence of anaemia reported by several authors in *T. vivax* infections in different hosts [33–37]. This anaemia might be due to haemolysis induced by the trypanosomes [38], or haemodilution arising as the fluid content of blood increases. Red cell counts can be reduced as a result of increased erythrophagocytosis, which was found to be an important mechanism leading to anaemia in the pathophysiology of *T. congolense* infection in Zambian goats [39], or of different immunological factors and dyshaemopoiesis in which the bone marrow fails to produce RBC [14, 40]. Haemolysis could also be caused by mechanical injury to erythrocytes by the lashing action of the powerful locomotory flagella and microtubule-reinforced bodies of the dense trypanosome population during elevated parasitaemia [41]. Erythrocyte membrane damage has also been postulated to be associated with adhesion of erythrocytes and reticulocytes to the trypanosome surface via sialic acid receptors leading to damage to erythrocyte cell membranes [42]. The significant increase in mean MCV value and insignificant change of mean MCH and MCHC indicate the macrocytic normochromic type of anaemia in infected groups, in agreement with Nadia *et al*. [43] and Gardiner [5]. Macrocytosis is due to erythrogenesis that takes place after the onset of infection, at which time immature erythrocytes are released into the bloodstream [44].

The reduction in total WBC count observed in this study was similar to that observed in other studies [32, 45, 46]. However, Nadia *et al*. [43] observed no significant difference in total WBC count in *T. vivax* infection in Sudanese Nubian goats. The lower counts of WBC, lymphocytes and neutrophils observed in the infected groups may be attributed to the immunosuppressive actions of trypanosome infection [47]. The increase in relative number of monocytes in infected groups may be explained by their role in the destruction of numerous damaged erythrocytes. In the present study, although body weight loss was lower in the NT groups than in the TT group, mainly the NT2 group was more severely affected by anaemia, indicating possibly inter-isolate variation in pathogenicity. Moreover, Shimelis *et al*. (unpublished data) demonstrated that the immunological response of the TT group was higher than that of the NT groups, which might demand excessive energy, at the expense of more body weight loss. Murray and Dexter [14] showed that the severity of anaemia during trypanosome infection could be related to differences in virulence among trypanosome strains.

## Conclusions

Young Zebu cattle infected with *T. vivax* isolates developed clinical trypanosomosis, which was associated with reduced feed intake, pyrexia, enlarged superficial lymph nodes, parasitaemia, emaciation, anaemia and leukopenia. *T. vivax* of a non-tsetse infested isolate (NT2) caused more severe anaemia than the tsetse infested *T. vivax* isolate, whereas the TT isolate resulted in much higher body weight loss than its NT counterparts. Therefore, the *T. vivax* parasite from non-tsetse infested area can at least be considered as important as that derived from the tsetse infested area.

## Abbreviations

MCH: Mean corpuscular haemoglobin
MCHC: Mean corpuscular hemoglobin concentration
MCV: Mean corpuscular volume
NIC: Non-infected control
NT1: Infected group with *T. vivax* isolate from non-tsetse infested area
NT2: Infected group with another *T. vivax* isolate from non-tsetse infested area
PCV: Packed cell volume
PID: Post infection day
RBC: Red blood cell
TT: Infected group with *T. vivax* isolate from tsetse infested area
WBC: White blood cell
